# Lactate-Loaded Nanoparticles Induce Glioma Cytotoxicity and Increase the Survival of Rats Bearing Malignant Glioma Brain Tumor

**DOI:** 10.3390/pharmaceutics14020327

**Published:** 2022-01-29

**Authors:** Víctor Chavarria, Emma Ortiz-Islas, Alelí Salazar, Verónica Pérez-de la Cruz, Alejandra Espinosa-Bonilla, Rubén Figueroa, Alma Ortíz-Plata, Julio Sotelo, Francisco Javier Sánchez-García, Benjamín Pineda

**Affiliations:** 1 Neuroimmunology and Neuro-Oncology Unit, Instituto Nacional de Neurología y Neurocirugía (INNN), Mexico City 14269, Mexico; vchavarriay2000@alumno.ipn.mx (V.C.); aleli.salazar@innn.edu.mx (A.S.); rubenfr@ciencias.unam.mx (R.F.); jsotelo@unam.mx (J.S.); 2 Laboratorio de Inmunorregulación, Departamento de Inmunología, Escuela Nacional de Ciencias Biológicas, Instituto Politécnico Nacional, Mexico City 11340, Mexico; 3 Nanotechnology Laboratory, Instituto Nacional de Neurología y Neurocirugía (INNN), Mexico City 14269, Mexico; emma.ortiz@innn.edu.mx; 4 Neurobiochemistry and Behaviour Laboratory, Instituto Nacional de Neurología y Neurocirugía (INNN), Mexico City 14269, Mexico; veped@yahoo.com.mx; 5 Central de Instrumentación, Posgrado en Inmunología, Escuela Nacional de Ciencias Biológicas, Instituto Politécnico Nacional, Mexico City 11340, Mexico; alecittab@gmail.com; 6 Experimental Neuropathology Laboratory, Instituto Nacional de Neurología y Neurocirugía (INNN), Mexico City 14269, Mexico; aortiz@innn.edu.mx

**Keywords:** lactate-loaded nanoparticle, glioma, metabolism, cytotoxicity, in situ therapy

## Abstract

A glioblastoma is an aggressive form of a malignant glial-derived tumor with a poor prognosis despite multimodal therapy approaches. Lactate has a preponderant role in the tumor microenvironment, playing an immunoregulatory role as well as being a carbon source for tumor growth. Lactate homeostasis depends on the proper functioning of intracellular lactate regulation systems, such as transporters and enzymes involved in its synthesis and degradation, with evidence that an intracellular lactate overload generates metabolic stress on tumor cells and tumor cell death. We propose that the delivery of a lactate overload carried in nanoparticles, allowing the intracellular release of lactate, would compromise the survival of tumor cells. We synthesized and characterized silica and titania nanoparticles loaded with lactate to evaluate the cellular uptake, metabolic activity, pH modification, and cytotoxicity on C6 cells under normoxia and chemical hypoxia, and, finally, determined the survival of an orthotopic malignant glioma model after in situ administration. A dose-dependent reduction in metabolic activity of treated cells under normoxia was found, but not under hypoxia, independent of glucose concentration. Lactated-loaded silica nanoparticles were highly cytotoxic (58.1% of dead cells) and generated significant supernatant acidification. In vivo, lactate-loaded silica nanoparticles significantly increased the median survival time of malignant glioma-bearing rats (*p* = 0.005) when administered in situ. These findings indicate that lactate-loaded silica nanoparticles are cytotoxic on glioma cells in vitro and in vivo.

## 1. Introduction

Glioblastoma (GBM) is the most frequent primary malignant tumor of the central nervous system in humans, with a dismal survival rate of 12–14 months despite the multimodal treatment currently used, which consists of surgical resection followed by chemo and radiotherapy [1].

Within the GBM microenvironment, a gradient of oxygen and nutrients that depends on the proximity to aberrant blood vessels has been found [2], functionally separating cells with an oxidative phenotype from cells with a glycolytic metabolism, yielding a heterogeneous tumor cell population [3] with metabolic adaptations that allow tumor cells to survive even under unfavorable conditions.

The metabolic adaptations generate a symbiosis between oxidative and glycolytic cells which relies on glycolysis as the main pathway for energy supply [4]. The progression of this pathway produces large amounts of lactate, reaching extracellular concentrations of up to 10–30 mM [5], in contrast with low intracellular concentrations. Lactate is secreted by tumor cells with high glycolytic capacity and is subsequently metabolized by oxidative tumor cells [6].

Lactate concentration is finely regulated by the activity of monocarboxylate transporters (MCT-1 and MCT-4) in the plasma membrane and by the enzymatic activity of lactate dehydrogenase (LDH), maintaining homeostatic levels. The modulation through the inhibition, blockade, or saturation of MCT-1, MCT-4, or LDH has been associated with tumor cell death in vitro, and reduced tumor growth in vivo [7,8]. The potential disruption of these therapeutic targets could be achieved by the local delivery of a metabolite that could distort this lactate-regulating system. It has been proven that the use of loaded nanocarriers in cancer therapy allows the delivery of drugs and molecules directly to the tumor milieu, with the capacity to alter the metabolic functions of tumoral cells [9]. As recently described by AbouAitah, mesoporous silica nanoparticles have great potential as carrier vehicles for molecules and drugs useful in cancer because of their high loading capacity and widespread biocompatibility [10].

Alteration in tumor metabolism is an emerging hallmark of cancer [11]. It has a direct impact on tumor dynamics, shaping its proliferative capacity, invasiveness, angiogenesis, and even the evasion of the antitumor immune response [12]. From the seminal description by Otto Warburg that aerobic glycolysis is the preferential mechanism of tumor cells to produce energy [13] to the description of a metabolic symbiosis between the different cell populations that constitute a solid tumor and its fine regulation at the molecular level [14,15,16], research clearly points to tumor cell metabolism as a therapeutic target.

We evaluated whether the administration of lactate at the intracellular level by lactate-loaded silica nanoparticles would decrease tumor cell viability in vitro and increase survival in an in vivo model of orthotopic malignant glioma. The use of this new lactate delivery vehicle is intended to be tested as a potential in situ adjuvant to current therapeutics for the management of patients with GBM.

## 2. Materials and Methods

### 2.1. Synthesis of Nanoparticles

The nanoparticles were synthesized in the Nanotechnology Laboratory at the Instituto Nacional de Neurología y Neurocirugía (Mexico City, Mexico), obtaining four substances: silica nanoparticles (SNP), lactate-loaded silica nanoparticles (LSNP), titania nanoparticles (TNP), and lactate-loaded titania nanoparticles (LTNP).

#### 2.1.1. Silica (SiO_2_) Nanoparticles

This synthesis was carried out based on the methodology previously described [17], using Pluronic P123 block copolymer (Sigma Aldrich, St. Louis, MO, USA) in an acid aqueous solution. Briefly, a solution of 4 g of Pluronic dissolved in 30 mL of deionized water was mixed with 100 mL of hydrochloric acid (0.2 M) (J.T. Baker, Phillipsburg, NJ, USA, 35.6–38%) solution at 35 °C; then, 466.5 mg of tetraethyl orthosilicate (Sigma Aldrich, 98%) was added and the solution was kept at 35 °C under stirring for 20 h. Finally, the resulting solid was washed several times with water, dried at 120 °C, and annealed at 550 °C. 

#### 2.1.2. Lactate-Loaded Silica Nanoparticles

The stirring/evaporation technique was used to load the silica nanoparticles with lactate in a 10:1 weight/weight ratio of silica/lactate. Briefly, 10 mg of lactate (Sigma Aldrich, Darmstadt, Germany) was dissolved in 20 mL of distilled water, and 100 mg of silica nanoparticles were added to this solution. The resulting mixture was left in stirring for 24 h at 35 °C. Finally, the solid obtained was dried at 70 °C for 12 h.

#### 2.1.3. Titania (TiO_2_) Nanoparticles

This sample was prepared by the sol-gel procedure from the hydrolysis-condensation of titanium tetrabutoxide, as previously described [18]. Briefly, 160 mL of ethanol was mixed with 80 mL of deionized water under agitation for 30 min at room temperature. Then, 10 mL with 9.7 g of titanium tetrabutoxide (Fluka, ≥97%) was added to the solution by dripping for 4 h under continuous agitation at 200 rpm at 35 °C. A homogeneous milky colloidal solution was gradually formed, and agitation was continued for 12 h. Afterward, the white precipitate was filtered and washed with deionized water and ethanol, and then it was dried at 70 °C for 12 h. From the initial 9.7 g of titanium tetrabutoxide, 2.2765 g of titanium dioxide was obtained, the base molecule of the nanoparticle.

#### 2.1.4. Lactate-Loaded Titania Nanoparticles

Lactate was used as the catalyst for the hydrolysis of titanium tetrabutoxide, so it would be part of the titania nanoparticle structure by generating covalent bonds. One gram of sodium lactate was dissolved in a mixture of 160 mL of ethanol and 80 mL of deionized water and kept under agitation for 30 min at room temperature. Then, 10 mL (9.7 g) of titanium tetrabutoxide was added to the solution by dripping for 4 h under continuous agitation at 200 rpm at 35 °C. When the homogeneous milky colloidal solution was formed, it was left under stirring for 12 h. Lastly, the precipitate was washed with deionized water and ethanol and dried at 70 °C for 12 h. The resulting product was TiO_2_ 2.2765:1 g lactate nanoparticles.

### 2.2. Characterization of Nanoparticles

The lactate load was confirmed by infrared light spectroscopy. Briefly, 5 mg (dry weight) of the nanoparticles were placed individually on the Perkin-Elmer GX spectrophotometer equipped with an ATR detector. One hundred measurements were obtained with a resolution of 8 cm^−1^, using Spectrum software for the acquisition of the samples. Results were analyzed using OriginPro 9 software.

The lactate release from nanoparticles was evaluated by a colorimetric reaction with FeCl_3_. Briefly, 500 µg of sterilized lactate-loaded silica nanoparticles were resuspended in 100 µL of DMEM (ThermoFisher Scientific, Waltham, MA, USA) without fetal bovine serum (FBS) and phenol red in triplicate, as well as 500 µg of LSNP in 100 µL of artificial cerebrospinal fluid (CSF), and stored at 37 °C. To perform dilution sampling, tubes were centrifuged at 14,000 rpm for 5 min and 5 µL of the supernatant was subsequently sampled at time points 0, 0.5, 1, 2, 2, 4, 6, 8, 24, 48, and 72 h. After each sampling, 5 µL of DMEM or CSF medium was added to the suspension to keep the total volume constant. Samples were stored at −20 °C until use.

Lactate quantification was determined by the colorimetric reaction with FeCl_3_ reported by Borshchevskaya et al. [19]. First, a standard curve was generated with lactate in a concentration range of 30 to 200 µg/mL in 0.01M NaOH, and the reaction solution was prepared with 0.3% FeCl_3_ dissolved in 0.01M HCl. Both solutions were mixed in a 1:1 volume ratio, incubated for 5 min, and absorbance was determined at 370 nm in a UV-Vis nanodrop spectrophotometer, using a 0.01M NaOH solution without lactate as a blank.

The samples were mixed with 0.02 M NaOH in a 1:1 volume ratio, and these were subsequently mixed with 0.3% FeCl_3_ 0.01M HCl solution in a 1:1 volume ratio, incubated for 5 min, and the absorbance was determined at 370 nm, using DMEM or lactate-free CSF as a blank. Finally, the lactate concentration was determined by linearly comparing the absorbance present with that reported in the standard curve, determining the rate of lactate release versus time. The same methodological approach was used to assess lactate release from lactate-loaded titania nanoparticles. The nanoparticle size distribution was measured in a Malvern Nanosight NS300 instrument (NanoSight, Amesbury, UK). Nanoparticles were suspended in PBS at a concentration of 250 µg/mL and sonicated for 30 min at room temperature (Branson 2210 Ultrasonic Bath). Readings were obtained using the red laser module (532 nm) and five 20 s recordings were generated for each nanoparticle sample. Analysis was carried out with the NTA 3.2 software.

Additionally, the size and shape of the nanoparticles were corroborated from transmission electron microscopy (TEM) images used for the ultrastructural analysis of the treated cells, as described below.

### 2.3. Glioma Cell Cultures

Rat malignant glioma C6 cells were obtained from the American Type Culture Collection (ATCC, Rockville, MD, USA). Sixty thousand cells per well were seeded in 24-well plates (Corning) with 1 mL of DMEM + 10% FBS (ThermoFisher Scientific, Waltham, MA, USA) + 100 U/mL of penicillin-streptomycin (Sigma Aldrich, Darmstadt, Germany) and kept in an incubator under sterile conditions at 37 °C in a humid environment with 5% CO_2_ until a 60% confluence was reached. Afterward, the cell cultures were treated with various nanoparticle concentrations (100 µg/mL, 50 µg/mL, and 25 µg/mL) for 72 h.

### 2.4. Ultrastructural Analysis of Nanoparticles by TEM

Briefly, after 24 h of treatment, C6 glioma cells were harvested by Trypsin/EDTA (Sigma Aldrich, Darmstadt, Germany), washed with PBS, and centrifuged at 2000 rpm for 5 min. The resulting pellet was fixed with 1 mL of 2.5% glutaraldehyde in 0.1 M cacodylate buffer pH 7.2 for 24 h. Afterward, the cells were centrifuged and washed only in cacodylate buffer from this point on, then post-fixed with 0.5% osmium tetroxide buffer for 1 h at room temperature. Cells were washed and dehydrated in increasing ethanol concentrations, starting with 70%, 80%, 90%, and 100% for 10 min each. Cells were suspended in propylene oxide for 10 min 2 times and pre-embedded in a 1:1 epoxy-propylene oxide resin mixture for 18 h at room temperature. Finally, the samples were embedded in epoxy resin, which was polymerized at 60 °C for 18 h. Semi-thin sections of 1 µm thickness were prepared with RMC ultramicrotome with a glass knife, the cuts were stained with toluidine blue and photographed under a NIKON microscope. Subsequently, ultrathin sections of 60 nm thickness were made with RMC ultramicrotome with a diamond-bladed knife. The slices were mounted on copper grids and kept in a humid chamber where the slices were contrasted with uranyl acetate for 20 min, washed with deionized water, then contrasted with lead citrate for 10 min and washed. The samples were observed and photographed in a Jeol transmission electron microscope (JEM 1400 PLUS).

### 2.5. Evaluation of Nanoparticle Uptake by Glioma Cells

After 24 h of treatment, nanoparticle-treated C6 cells were harvested by Trypsin/EDTA, washed twice, resuspended in PBS, and analyzed in a FACSCalibur flow cytometer (10,000 cumulative events were analyzed by CellQuest Pro software, Franklin Lakes, NJ, USA). The mean cell granularity was obtained (side scatter, SSC). Results were plotted as mean increase ± SEM from three different experiments made by triplicate. For the calculation of the percentage change in granularity, the mean event granularity was obtained, and the data were normalized in respect to the control considered as 100%. Finally, 100 was subtracted from each value to obtain the net percentage increase. Additionally, the amount of NP uptake by the glioma cells was assessed using a CytoFlex SRT nano-flow cytometer and violet laser (Beckman Coulter, Indianapolis, IND, USA). Briefly, C6 and RG2 cells were treated for 24 h with 100 µg/mL SNP; next, the cell supernatants were collected. The Cytoflex SRT settings were adjusted by using a nano fluorescent particle size standard kit (Spherotech, Lake Forest, IL, USA), and the number of events in each sample was assessed using the same parameters, at the same flow rate, and for the same length of acquisition time (3 min). The amount of NPS endocytosed by glioma cells was calculated by subtracting the number of nanoparticles in the cell supernatants from the number of nanoparticles in the NP stock of 100 µg/mL.

### 2.6. Evaluation of Cellular Metabolic Activity

Dehydrogenase-dependent metabolic activity was evaluated by a 3-(4,5-dimethyltiazol-2-yl)-2,5-diphenyltetrazolium bromide (MTT) (Sigma Aldrich, Darmstadt, Germany) reduction assay. After 72 h of treatment, the medium was removed and C6 cells were washed with PBS, then 100 µL of MTT to a concentration of 5 mg/mL in DMEM was added to each well and incubated for 4 h at 37 °C. Afterward, the medium was aspirated, and blue formazan salts were solubilized with 100 µL of acidic isopropanol. The quantification of formazan was determined by optical density at a wavelength of 570 nm in a plate reader (EON, BioTek, Winooski, VT, USA), the results were analyzed using the Gen5 program. Data were obtained from three independent experiments by triplicate.

### 2.7. Evaluation of Cytotoxicity

After 72 h, cells were harvested, washed, and resuspended in PBS with propidium iodide (PI) (Sigma Aldrich, Darmstadt, Germany) to a concentration of 5 µg/mL and incubated for 15 min in the dark. Then, 10,000 events were acquired in a FACSCalibur flow cytometer. Analysis was performed using CellQuest Pro and FlowJo v10 software. Data were obtained from three independent experiments performed in triplicate.

### 2.8. Evaluation of Cell Supernatant Acidification

Sixty thousand cells per well were seeded in 24-well plates (Corning, Corning, NY, USA) with 1 mL of DMEM+ 10% FBS and treated with 100 µg/mL of SNP, LSNP, TNP, and LTNP. We obtained 1 mL of the supernatant at 24, 48, 72, and 96 h after treatment. pH measurements were made with a Beckman 41 potentiometer. Data were obtained from three independent experiments performed in triplicate.

Then, the pH of the culture medium with resuspended nanoparticles was measured as a control. Briefly, 1 mL of DMEM per well was used in 24-well plates, and 100 µg/mL of SNP, LSNP, TNP, and LTNP were added and kept at room temperature in continuous agitation. One mL samples were obtained at 30, 60, 120, 180 min, 6, 24, 48, and 72 h, and preserved at 4 °C until measurement. pH measurements were made with a Beckman 41 potentiometer. Data were obtained from three independent experiments performed in triplicate.

### 2.9. Evaluation of Cellular Metabolic Activity under Hypoxia

The effects of nanoparticle treatment on C6 cells were evaluated under hypoxia and different glucose concentrations to mimic the different nutrient dispositions in a solid tumor. CoCl_2_ (320 µM) was used to induce chemical hypoxia [20], and cells were cultured with three different glucose concentrations in media: 5.5 mM, 17 mM, and 25 mM. Briefly, 6,000 cells were seeded in 96-well plates and treated with 100 µg/mL of SNP, LSNP, TNP, and LTNP. A lactate-added control group was also included (10 µg/mL). After 72 h, we performed an MTT reduction assay to evaluate metabolic activity.

### 2.10. Evaluation of Cytotoxicity on Cocultured Normoxic and Hypoxic Glioma Cells

To mimic the interaction between normoxic and hypoxic cells found within a solid tumor such as GBM, we performed a coculture of hypoxic and normoxic-conditioned C6 cells and evaluated the cytotoxicity induced by nanoparticle treatment. Normoxic C6 cells were conditioned to a culture medium with glucose (5.5 mM) (DMEM low-glucose, ThermoFisher Scientific, Waltham, MA, USA) and lactate (32 mM) for 3 weeks. Hypoxic C6 cells were cultured with the addition of glucose (25 mM) (DMEM high-glucose, ThermoFisher Scientific, Waltham, MA, USA) + CoCl_2_ (320 µM) for 3 weeks.

Briefly, we stained normoxic-conditioned C6 cells with CFSE (1:1000) and mixed them with hypoxic-conditioned C6 cells (1:1 ratio). Sixty thousand cells were seeded in 24-well plates and kept in darkness until 60% confluence, then treated with 100 µg/mL of SNP, LSNP, TNP, and LTNP. After 72 h, cells were harvested and stained with PI for flow cytometry analysis, as previously described. Cell populations were separated by CFSE positivity. Data were obtained from two independent experiments performed in triplicate.

### 2.11. Evaluation of the Effects of Nanoparticles in an In Vivo Orthotopic Malignant Glioma Model

Animal care and the use of all experimental animals were performed in accordance with institutional ethical guidelines. All animal experiments were approved by the Bioethics Committee and the Institutional Review Board (Number 11/19) of the National Institute of Neurology and Neurosurgery and followed the regulations of the Mexican Standard for the production, care, and use of laboratory animals (NOM-062-ZOO-1999). We used 51 male Wistar rats weighing 150–180 g, maintained with food and water ad libitum, and separated into 5 groups: (1) control, (2) SNP, (3) LSNP, (4) TNP, and (5) LTNP.

Rats were anesthetized by the intraperitoneal administration of Zoletil-50 (30 mg/kg) and Procin (8 mg/kg). A cavity was made (Mini Electrodrill LS-100) at 2 mm anterior × 2 mm lateral-right to the bregma. C6 cells were harvested, counted, and adjusted to 10^6^ cells/10 µL DMEM, then 10 µL of the C6 cell suspension was introduced 3 mm through the dura, inoculating for 5 min.

One week after the implant, the surviving animals were treated as follows: group 1 was intratumorally injected with 10 µL of PBS, and groups 2, 3, 4, and 5 were intratumorally injected with 1 mg /10 µL of the corresponding suspension of nanoparticles. Survival time was assessed from the date of tumor implantation.

### 2.12. Statistical Analysis

Data were expressed as the mean ± standard error of the mean (SEM). GraphPad Prism 6 software was used for statistical analysis as follows: one-way ANOVA test with Dunnett multiple comparison test and 95% confidence interval (CI) for nanoparticle internalization, MTT reduction assay, and cell death evaluation; two-way ANOVA test with Dunnett’s multiple comparison test and 95% CI for pH measurement of DMEM with nanoparticles and supernatant pH of treated cells. Cumulative survival was determined from the day of implantation until death. The data were plotted by a Kaplan–Meier curve with the log-rank test.

## 3. Results

### 3.1. Nanoparticle Characterization

**Figure 1:**
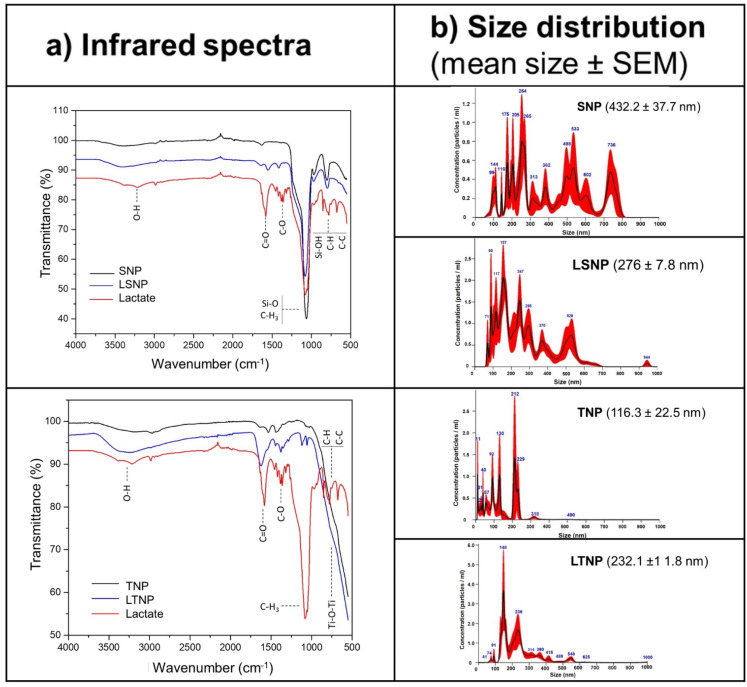
Silica and titania nanoparticles were efficiently loaded with lactate. (**a**) Five mg of each (dry weight) of the nanoparticles were analyzed on a spectrophotometer with an ATR detector, obtaining the infrared spectra of the silica nanoparticles (SNP-black line) and the lactate-loaded silica nanoparticles (LSNP-blue line) compared with the lactate spectrum (red line) (upper left panel), and the infrared spectra of the titania nanoparticles (TNP-black line) and the lactate-loaded titania nanoparticles (LTNP-blue line) compared with the lactate spectrum (red line) (lower left panel). (**b**) Nanoparticles were suspended in PBS at 250 µg/mL and analyzed with the red laser module (532 nm) of the Malvern Nanosight NS300 instrument. Histograms show mean particle size distribution, the black line represents mean size, and red bars show ±1 SEM.

Once silica and titania nanoparticles with and without lactate loading were synthesized, their attributes were characterized. First, an infrared spectroscopy assay was performed to determine their composition and corroborate the presence of lactate in the nanoparticles. The upper left panel in Figure 1a shows the infrared spectrum silica-based nanoparticles. The SNP-infrared spectrum shows the classical vibrations that characterize the silica structures. The silanol (Si-OH) and siloxane (Si-O) groups are located at 800 cm^−1^ and 1100 cm^−1^, respectively. The band observed around 3100–3700 cm^−1^ comes from O-H bonds. The lactate infrared spectrum presented bands at 500–900 cm^−1^, corresponding to C-H and C-C bonds, as well as a predominant peak at 1100 cm^−1^ originated by C-H_3_ bonds. The bands at 1300 cm^−1^ and 1650 cm^−1^ were formed from the C-O and C=O bonds present in the lactate structure, respectively. The band at 3000–3500 cm^−1^ was derived from hydroxyl groups (OH). When comparing the LSNP spectrum to the SNP spectrum, we found additional bands at 1300 cm^−1^ and 1650 cm^−1^, that correspond to C-O and C=O bonds, suggesting that LSNP were successfully loaded with lactate.

The TNP spectrum showed a stretching band at 500–900 cm^−1^ determined by Ti-O-Ti bonds, while the band around 3100–3700 cm^−1^ corresponds to hydroxyl (O-H) surface groups. In the spectrum of LTNP, bands at 1100 cm^−1^, 1300 cm^−1^, and 1600 cm^−1^ are observed, which are given by C-H_3_, C-O, and C=O bonds, respectively. The wide band located at 3000–3700 cm^−1^ is related to O-H bonds. The observation of these bands suggests that LTNP were successfully loaded with lactate, as shown in the lower-left panel in Figure 1a.

A particle size distribution analysis showed that SNP have an average size of 432.2 ± 37.7 nm, however, they were the most heterogeneous type of nanoparticles, as seen in Figure 1b. LSNP had a mean size of 276 ± 7.8 nm. In the case of TNP, they showed a mean size of 116.3 ± 22.5 nm, and 232.1 ± 11.8 nm for the LTNP. Overall, titania-based nanoparticles had smaller sizes compared to silica-based nanoparticles.

### 3.2. Cumulative Lactate Release from Nanoparticles

**Figure 2:**
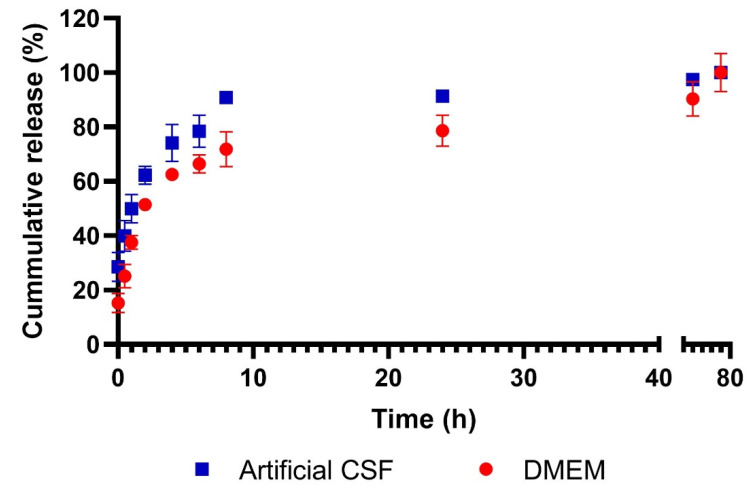
Cumulative lactate release from LSNP. Nanoparticles were resuspended in DMEM and artificial CSF, obtaining samples over 72 h. The percentage of lactate released was calculated by spectrophotometry with FeCl_3_ reaction assay. The graph shows the mean ± SEM of 3 independent experiments in triplicate in artificial CSF (blue) and DMEM (red).

The profile of lactate release from silica nanoparticles was evaluated in DMEM and in artificial cerebrospinal fluid (CSF). Lactate release occurred in both DMEM and CSF with similar behavior; a 60–70% release of the load was observed in the first 4 h, followed by a sustained release that was completed at 72 h, as shown in Figure 2. Otherwise, lactate release from LTNP was neither observed in DMEM nor CSF over 72 h (Figure not shown).

### 3.3. Nanoparticle Treatment Induces Intracellular Accumulation of Nanometer-Sized Electron-Dense Particles

**Figure 3:**
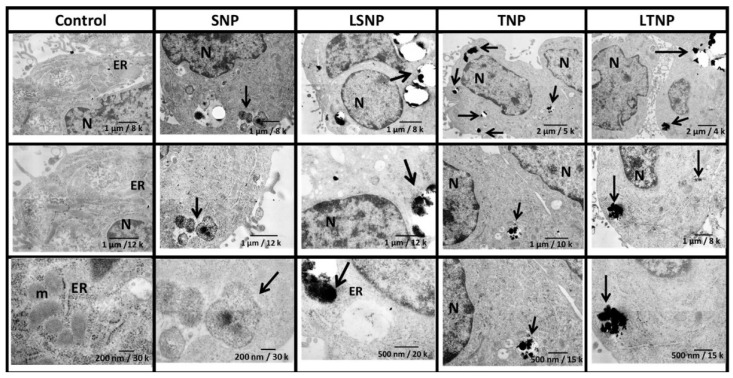
Nanoparticle treatment induces the intracellular accumulation of nanometer-sized electron-dense granules. C6 cells were treated with 100 μg/mL of nanoparticles for 24 h, fixed in glutaraldehyde, and processed for visualization by transmission electron microscopy. Representative images of cells are stocked in columns from lower to higher magnification. Key: endoplasmic reticulum (ER), nucleus (N), mitochondria (m), nanoparticles (arrows).

The ultrastructure of nanoparticles and their intracellular localization were evaluated by TEM. Non-treated control cells show normal ultrastructural features (Figure 3). Overall, treated cells display membrane-surrounded nanometric electron-dense intracellular granules (nanoparticles). In SNP-treated cells, the nanoparticles display a porous spherical shape with an empty appearance and a diameter of approximately 400 nm. In contrast, LSNP-treated cells present spherical nanoparticles with less evident but more electron-dense pores due to the lactate loading inside the nanoparticle pore. Variable sizes could be observed. TNP-treated cells present non-porous amorphous nanoparticles, with sizes ranging from 100 to 200 nm, and a tendency to form aggregates into vesicles. Similar shapes and sizes could be observed as in the case of LTNP.

### 3.4. Nanoparticle Treatment Increases Cell Granularity of C6 Glioma Cells

**Figure 4:**
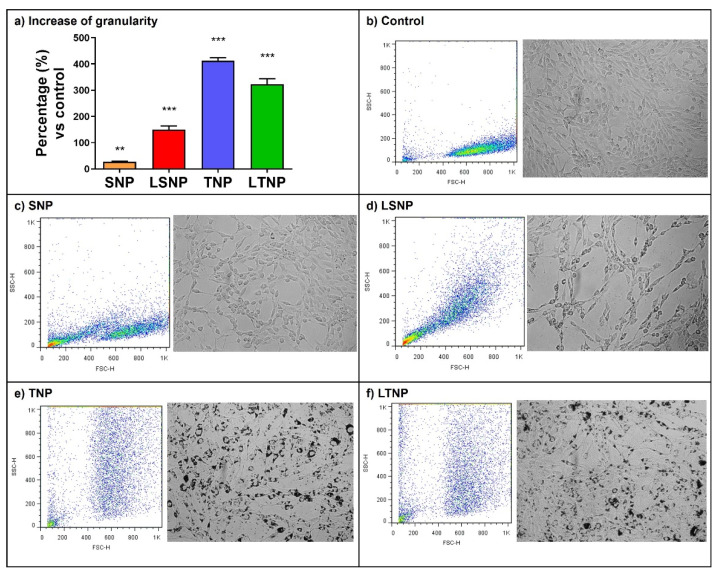
Nanoparticle treatment increases the granularity of C6 cells. Cells were treated with 100 μg/mL of nanoparticles for 24 h and analyzed by flow cytometry, obtaining the mean granularity. (**a**) Percentage increase in the granularity of treated cells, bars show mean ± SEM of three independent experiments in triplicate, *n* = 9 (statistical analysis with one-way ANOVA after logarithmic transformation, ** *p* < 0.001 and *** *p* < 0.0001 vs. control). Representative dot plots (left) and bright-field images (right) of treated C6 cells: (**b**) untreated control, (**c**) SNP, (**d**) LSNP, (**e**) TNP, (**f**) LTNP.

The internalization of nanoparticles was determined by the percentage increase in cellular granularity. All nanoparticles induced a statistically significant increase in granularity versus control, SNP treatment generated a 26% increase (*p* < 0.001), while LSNP treatment shows a 179% increase (*p* < 0.0001). The greatest increase was found in TNP-treated cells with 439% (*p* < 0.0001) and LTNP with 378% versus the control (*p* < 0.0001), as shown in Figure 4. All the NPs were uptaken by the C6 glioma cells by more than 80% after 24 h of treatment (Supplementary Figure S1). Overall, the nanoparticles show a tendency to aggregate in the cytoplasm of tumor cells.

### 3.5. The Cell Metabolic Activity Decreases in a Dose-Dependent Manner after Treatment

After finding that C6 glioma cells internalize the nanoparticles efficiently, we evaluated the effects of nanoparticles on tumor cells. First, we evaluated the cellular metabolic activity dependent on dehydrogenases, finding that treatment with each of the nanoparticle groups reduced the dehydrogenase-dependent metabolic activity of C6 cells in a dose-dependent manner, as assessed by the MTT reduction assay, suggesting that the nanoparticles had an intrinsic biological effect, regardless of their chemical composition and whether they carried lactate.

**Figure 5:**
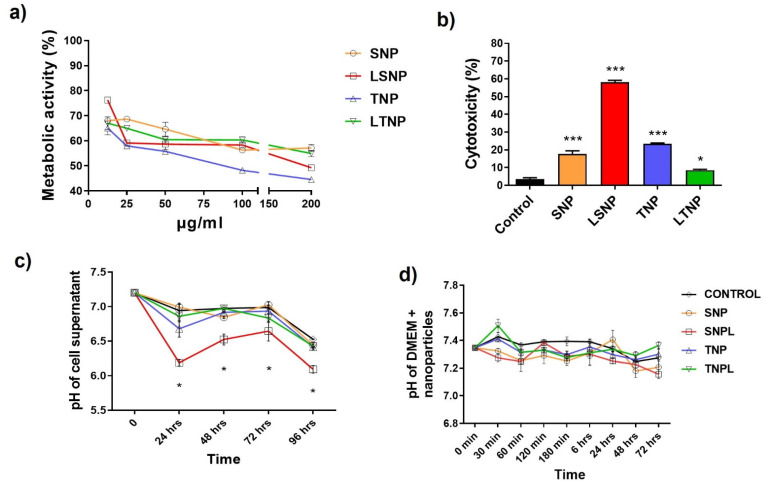
Nanoparticle treatment induces a decrease in metabolic activity in a dose-dependent manner, and LSNP are highly cytotoxic and induce the acidification of the cellular supernatant. C6 cells were treated with 100 μg/mL of nanoparticles for 72 h. (**a**) Dehydrogenase-dependent metabolic activity was measured by MTT reduction assay. Lines show mean percentage vs. control ± SEM (n = 6). (**b**) Cytotoxicity was measured by propidium iodide staining. Bars show the mean percentage of dead cells ± SEM (n = 9). (**c**) The supernatant of treated cells was collected for pH measurement. Lines show mean pH value ± SEM over 96 h (n = 6). (**d**) Nanoparticles were resuspended in a culture medium for pH measurement. Lines show mean pH value ± SEM over 72 h (n = 6); (statistical analysis with one-way ANOVA after logarithmic transformation for cytotoxicity evaluation, and two-way ANOVA for pH measurement: * *p* < 0.05 and *** *p* < 0.0001 vs. control).

LSNP and TNP treatment resulted in a higher decrease in metabolic activity at lower doses (Figure 5a). Interestingly, LSNP at doses of 25 and 50 μg/mL were found to have a significantly greater effect when compared to SNP (*p* ≤ 0.001), so the loading of lactate probably generated a greater effect on C6 cells.

### 3.6. LSNP Are Highly Cytotoxic and Accompanied by Supernatant Acidification

We then evaluated the cytotoxicity on C6 tumor cells to determine whether the nanoparticles would have antitumor effectiveness, finding that LSNP generated 58.1% of dead cells after 72 h of treatment, significantly higher compared to the control and the other nanoparticles (*p* < 0.0001). SNP caused 17.6% cytotoxicity (*p* < 0.0001 vs. control/LSNP), suggesting that LSNP cytotoxicity is dependent on the loaded lactate. Interestingly, TNP induced 23.4% cytotoxicity (*p* < 0.0001 vs. control/LTNP) and was higher when compared to LTNP cytotoxicity (8.5% with *p* < 0.05 vs. control) (Figure 5b).

The culture medium had a basal pH of 7.2, while the pH decreased to 6.9 on average after 24 h. However, LNPS treatment induced a more pronounced acidification (pH 6.2) which was maintained throughout 96 h when compared to the control (*p* < 0.0001). The greater acidification of the supernatant was likely attributed to the cytotoxic effect of LSNP (Figure 5c). Figure 5d shows that SNP, as well as LSNP, TNP, and LTNP suspended only in a culture medium did not achieve significant pH modifications over 72 h. These findings allow us to link the greater acidification of the supernatant to the direct effect of the nanoparticles on tumor cells.

### 3.7. Decrease in Cell Metabolic Activity Induced by Nanoparticles Is Inhibited during Hypoxia

**Figure 6:**
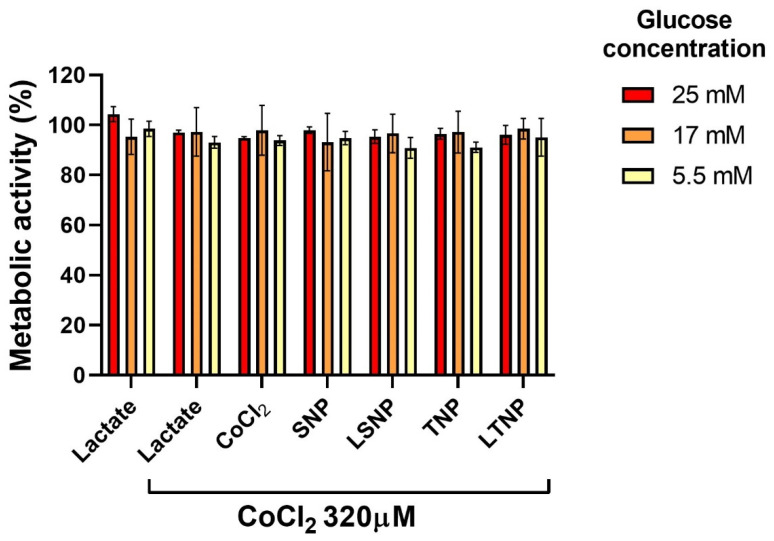
The decrease in cell metabolic activity induced by nanoparticles is inhibited during hypoxia. C6 cells were cultured under chemical hypoxia with 320 µM of CoCl_2_ and a glucose concentration of 5.5 mM, 17 mM, and 25 mM and treated with 100 μg/mL of nanoparticles for 72 h. Dehydrogenase-dependent metabolic activity was measured by MTT reduction assay. Bars show mean percentage vs. control without CoCl_2_ ± SEM of three independent experiments in triplicate, n = 9 (statistical analysis with one-way ANOVA after logarithmic transformation).

As shown in Figure 6, the addition of lactate (10 mg/mL) to the control cells did not alter the cellular metabolic activity, neither in normoxia and hypoxia nor at different glucose concentrations in a culture medium. Dehydrogenase-dependent metabolic activity was not significantly modified after the treatment with 100 μg/mL of any of the nanoparticles. This inhibition of decrease in metabolic activity occurred independently of glucose concentration, as the results were similar for cells cultured with culture media containing 5.5 mM, 17 mM, and 25 mM glucose.

The above findings indicated that, under hypoxia, there are no nanoparticle-induced alterations in metabolic activity, independent of glucose availability.

### 3.8. LSNP-Induced Cytotoxicity Is Inhibited by the Interaction between Normoxic and Hypoxic Tumor Cells

**Figure 7:**
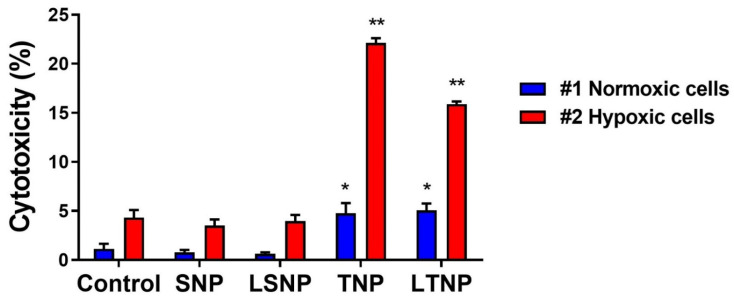
LSNP-induced cytotoxicity is inhibited by the interaction between metabolically conditioned tumor cells. C6 cells were cultured under normoxia and hypoxia for 3 weeks, normoxic cells (glucose 5.5 mM + lactate 32mM, blue bars) and hypoxic cells (glucose 25mM + CoCl_2_ 320 µM, red bars), and then co-cultured and treated with 100 μg/mL of nanoparticles for 72 h for the evaluation of cytotoxicity with propidium iodide staining. Bars show mean percentage of dead cells ± SEM of two independent experiments in quadruplicate, n = 8 (statistical analysis with one-way ANOVA after logarithmic transformation, * *p* < 0.001 and ** *p* < 0.0001 vs. their respective untreated controls).

To mimic the interaction between normoxic and hypoxic cells found within a solid tumor such as GBM, we performed a coculture of hypoxic and normoxic-conditioned C6 cells and evaluated the cytotoxicity induced by nanoparticle treatment. As shown in Figure 7, TNP induced the highest cytotoxicity (>20%), predominantly in hypoxic C6 cells (*p* ≤ 0.0001 vs. control). LTNP were less cytotoxic than TNP, consistent with previous results (*p* < 0.0001 vs. TNP). Both TNP and LTNP were discretely—but significantly—cytotoxic against normoxic C6 cells (*p* < 0.001 vs. #1 control). Surprisingly, LSNP did not show a significant cytotoxic effect on any of the cell populations in co-culture. These data suggest that the interaction between co-cultured tumor cells with different metabolic conditioning is a key determinant of the cytotoxic capacity of LSNP.

### 3.9. LSNP Treatment Increases Median Survival in Model of Orthotopic Malignant Glioma

**Figure 8:**
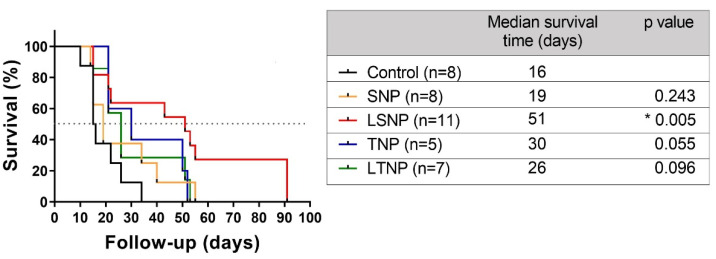
In situ administration of lactate-silica nanoparticles increases the median survival time in rats bearing orthotopic malignant glioma. Stereotactic implantation of 1 × 10^6^ C6 cells in the right cerebral hemisphere of male Wistar rats; after 7 days they were treated with the intratumoral administration of 1 mg of nanoparticles. Survival follow-up of rats surviving the surgical procedure. Data are shown in a Kaplan–Meier survival curve (statistical analysis by log-rank test, * *p* = 0.005).

Finally, to determine the therapeutic efficacy of nanoparticle treatment in an in vivo model, nanoparticles were administered in situ in a model of orthotopic malignant glioma. Figure 8 shows in the Kaplan–Meier curve that LSNP-treated tumor bearing-rats had a statistically significant increase in the median survival time with 51 days when compared to the control with 16 days (*p* = 0.005), demonstrating the therapeutic efficacy of in situ administration of LSNP. Other nanoparticles did not show statistically significant differences with respect to the control group.

## 4. Discussion

Currently, GBM is a deadly brain tumor, despite multimodal therapy. Related to the metabolic features of GBM that promote its malignant behavior, these features could be addressed in therapy to improve the prognosis.

We propose that the delivery of a lactate overload carried in nanoparticles, allowing the intracellular release of lactate, would compromise the survival of tumor cells. This work describes the synthesis and physicochemical characterization of silica and titania nanoparticles loaded with lactate, as well as their effect on the viability of rat glioma C6 cells under different metabolic conditions, based on the hypothesis that when lactate is released from the nanoparticles into the cell cytoplasm, it would alter the lactate homeostasis, leading to tumor cell death. The most relevant finding is that lactate-loaded silica nanoparticles exert a major cytotoxic effect on C6 cells and improve survival time in the orthotopic malignant glioma model.

In this study, functionalized silica nanoparticles were synthesized through the template method [17], characterized by the capability to form hydrogen bonds, which would act as efficient carriers of molecules such as lactate. For the synthesis of functionalized covalently bonded lactate-titania nanoparticles, the precursor titanium tetrabutoxide was used, having highly reactive electronegative alkoxy groups, and then lactate was added to catalyze the hydrolysis of the alkyl groups, generating covalent bonds with the resulting titanium dioxide, containing a high concentration of surface hydroxyl groups. The infrared spectrum of SNP agrees with previous reports [21], as well as for TNP [22], and importantly, the presence of lactate in both LSNP and LTNP was corroborated by the IR spectra.

Since the LSNP were loaded with lactate via non-covalent bonds, it was expected that lactate would be released from the nanoparticles over time, so the lactate release profile was analyzed via a FeCl_3_-dependent colorimetric reaction, showing an initial rapid release of lactate during the first hours in both DMEM and artificial CSF, with a subsequent slowing and sustained release over 72 h. Multiple drug release assays from silica nanoparticles have been reported, finding similar behaviors [23]. Recently, Ortiz-Islas et al. reported the use of cisplatin-loaded silica nanoparticles focused on the management of glioblastoma, showing a 50% drug release in the first 4 h in artificial CSF, correlating to our work [24].

The C6 cell line is considered an adequate experimental model for GBM research, as C6-derived orthotopic tumor models show a tumoral behavior with high homology to GBM, possessing a high proliferative rate of pleomorphic cells with infiltrative capacity, neoangiogenesis, and the formation of necrotic areas [25].

When C6 cells were exposed to nanoparticles in vitro, an increase in granularity was induced, as a reflection of their internalization [26]. Both SNP [27] and TNP [28] have been reported to induce increased granularity as measured by flow cytometry. Previous reports from our work group point out the importance of nanoparticle functionalization as a determinant of bioavailability; when nanoparticles are functionalized, they can be endocytosed by tumor cells more effectively [29]. In our study, nanoparticles were identified as electron-dense granules in TEM images, and they were mostly contained inside of membranous organelles as endosomes, showing a tendency to form aggregates. The main mechanism used for the internalization of silica and titania nanoparticles is caveolin-mediated endocytosis; however, direct diffusion through the cell membrane has also been reported, improving direct access to the cytosol [30]. The shape and size of nanoparticles observed by TME were in concordance with the synthesis and Nanosight analysis. In addition, LSNP were found to show more electron-dense pores due to the loaded lactate.

The next step of our study was to assess the effects of nanoparticles on C6 tumor cells. We first evaluated the impact on metabolic activity through the MTT reduction assay. The MTT reduction assay continues to be the basis for many in vitro studies to evaluate the anticancer activity of new drugs. Besides its conventional use as an indicator of cell viability, this assay reflects dehydrogenase-dependent metabolic activity [31,32]. Here, it was found that C6 cells treated with the different nanoparticles show a dose-dependent decrease in metabolic activity dependent on dehydrogenases, probably due to metabolic stress caused by the direct presence of the nanoparticles.

Treatment with LSNP produced the highest cytotoxicity, correlating with significant and persistent supernatant acidification, probably due to cell death induced by the release of lactate, leading to intracellular lactate overload, which could impair the progression of the metabolic pathways involved in its generation and clearance [33,34]. The induction of oxidative stress in C6 cells impairs their ability to synthesize ATP, leading to the cessation of ATP-dependent proton pump activity, thus acidifying the intracellular compartment [35,36]. When these mechanisms are maintained, cell death occurs with the release of protons that acidify the extracellular medium. The cytotoxic effect of LSNP attributed to lactate release could be dependent on the expression of lactate transporters as well as on the allosteric inhibition of glycolysis enzymes. In this regard, the constitutively expressed MCT1 transporter has a Km of 4 mM for lactate, showing saturation at higher lactate concentrations. In contrast, the expression of the MCT4 transporter, which is hypoxia-induced has a Km for lactate close to 30 mM [37,38]. Therefore, the lactate release from endocytosed LSNP is expected to increase the intracellular lactate concentration, saturating the activity of MCT1 transporters from within. In the same scenario, hypoxic cells would be expected to be resistant to the increase in intracellular lactate, due to the upregulation of MCT4 transporters. In this study, the lactate-loaded nanoparticles suspended only in culture medium were not sufficient to generate a significant pH change, since lactate acts as a weak base in its ionized form, then is buffered when dissolved in an aqueous medium [39].

TNP also induced cytotoxicity and a reduction in metabolic activity, which was significantly lost when TNP were bound to lactate, likely due to increased biocompatibility, as has been described for poly-lactic acid-functionalized titanium dioxide nanostructures [40]. We identified that lactate covalently bound to TNP made them more biocompatible, and that lactate loaded on SNP generated a greater cytotoxic and metabolic disrupting effect as compared to SNP alone.

Based on these results, we tested some metabolic scenarios that can be found within a solid tumor. Thus, C6 cells were cultured with CoCl_2_, known to antagonize prolyl hydroxylases, which would stabilize HIF1α and induce chemical hypoxia. As a consequence, tumor cells will express a glycolytic phenotype [41]. Under this hypoxic scenario, nanoparticle treatment did not induce any modification of cellular metabolic activity, and this seems to be independent of the glucose concentration.

Additionally, we cultured C6 cells with a low glucose concentration and high lactate availability and kept them in normoxia; simultaneously, we cultured cells exposed to CoCl_2_ and a high glucose concentration, so their metabolism could theoretically lean towards an oxidative and glycolytic phenotype, respectively. These characteristics were used to recreate the metabolic symbiosis when co-cultured. Strikingly, LSNP treatment did not result in cell death in either cell population, which may be due to the direct interaction between these metabolically conditioned cells.

Previous reports have compared the behavior of pancreatic adenocarcinoma [42,43] and colon adenocarcinoma [44] tumor cells in co-culture with fibroblasts, monocytes, or normal colon cells, generating a pro-tumoral microenvironment with immunosuppressive capacity and resistance to chemotherapeutics. Moreover, in vitro studies with glioma cell lines (A172, U251, LN18, C6) in co-culture with astrocytes have shown increased resistance to drugs such as temozolomide and doxorubicin [45].

Few studies have addressed metabolic modifications on cells in co-culture. Evidence shows that the co-culture of lung carcinoma cells or pancreatic carcinoma cells with fibroblasts promotes an oxidative phenotype in tumor cells [46,47]; in contrast, cervical carcinoma cells increase their glycolytic phenotype [48]. However, there are no studies where cells with different metabolic phenotypes are maintained in co-culture, to explain why the cytotoxicity exerted by LSNP was inhibited in this model.

Since the median survival of patients diagnosed with GBM is short, increased survival has been used as an indicator of treatment efficacy [49]. We found that LSNP treatment considerably increased the median survival time of rats, probably related to its effect on inducing cytotoxicity in tumor cells. The amount of lactate contained in the nanoparticles that were injected within the rat tumors is equivalent to 23 times (111.11 mM) the lactate concentration reported within the glioblastoma tumors measured by Magnetic Resonance Spectroscopic imaging (4.77 mM range 1.5–9.2 mM). On the other hand, lactate is not detected in normal-appearing white matter [50], so it appears that the concentration that we injected into the rat tumors is clinically relevant and, therefore, that it is possible to alter the lactate concentration within the tumor mass, inducing tumor cell death. Various metabolic disruption approaches have been reported: McKelvey et al. have recently shown that the inhibition of glycolysis and fatty acid oxidation leads to a modest but significant increase in the survival of orthotopic murine models of GBM [51]. Another study using the lactate transporter inhibitor, α-cyano-4-hydroxy-cinnamic acid, with continuous intratumoral infusion in GBM orthotopic xenograft in nude rats demonstrated a twofold increase in the median survival time by increasing the intracellular concentration of lactate [52]. Wicks et al., using a local delivery device wafer loaded with 3-bromopyruvate plus temozolomide and radiotherapy, found a synergic effect, obtaining a longer overall survival [53]. Liang et al. assessed the intravenous administration of albendazol-silver nanoparticles on C6 malignant glioma-bearing nude mice; such nanoparticles have been reported to have glycolytic and mitochondrial inhibition properties, yielding an increase of 9 days in median survival time [54].

The alteration of cancer cells from a metabolic perspective is an attractive therapeutic target that could be addressed through nanomedicine. Having shown therapeutic efficacy, as future perspectives derived from this work, we are working on evaluating the mechanism of action of nanoparticles on tumor cells, as well as the impact of the cytotoxicity of nanoparticles on the activation of the antitumor immune response.

## 5. Conclusions

Our research shows that the topical administration of LSNP in a model of orthotopic malignant glioma increases survival by inducing cytotoxicity on tumor cells, thus having the potential to be a promising adjuvant tool for the treatment of solid tumors such as glioblastoma.

## Data Availability

The data presented in this study are available on request from the corresponding author.

## Supplementary Materials

The following supporting information can be downloaded at: https://www.mdpi.com/article/10.3390/pharmaceutics14020327/s1, Figure S1: Cellular uptake of SNP by glioma cells. C6 and RG2 glioma cells were incubated for 24 h with 100 μg/mL SNP; next, cell supernatants were collected. The amount of NPS endocytosed by glioma cells was calculated by subtracting the amount of nanoparticles in the cell supernatants from the amount of nanoparticles in the stock of 100 μg/mL. Nanoparticles counting was performed using a CytoFlex SRT cell sorter and its violet laser (Beckman Coulter, USA). Figure shows representative dot plots of injectable water (**A**), calibration control beads for the violet laser (**B**), 100 μg/mL SNP stock (**C**), supernatant of treated C6 cells (**D**) and supernatant of treated RG2 cells (**E**). Percentage of NPs uptake by C6 and RG2 cells. Results are presented as mean ± SD of one experiment by triplicate.
