# Supplementary material for: Lactate-Loaded Nanoparticles Induce Glioma Cytotoxicity and Increase the Survival of Rats Bearing Malignant Glioma Brain Tumor
Source: Pharmaceutics. 2022 Jan 29;14(2):327. doi: 10.3390/pharmaceutics14020327 (PMC8880216; doi:10.3390/pharmaceutics14020327)
Supplement: Supplementary file 1 [file pharmaceutics-14-00327-s001.zip › pharmaceutics-1532572-supplementary.pdf]

pharmaceutics

MDPI

Supplementary File

Lactate loaded nanoparticles induce glioma cytotoxicity and increase survival of rats bearing malignant glioma brain tumor.

Víctor Chavarria, Emma Ortiz-Islas, Alelí Salazar, Verónica Pérez-de la Cruz, Alejandra Espinosa-Bonilla, Rubén Figueroa, Alma Ortíz-Plata, Julio Sotelo, Francisco Javier Sánchez-García and Benjamín Pineda

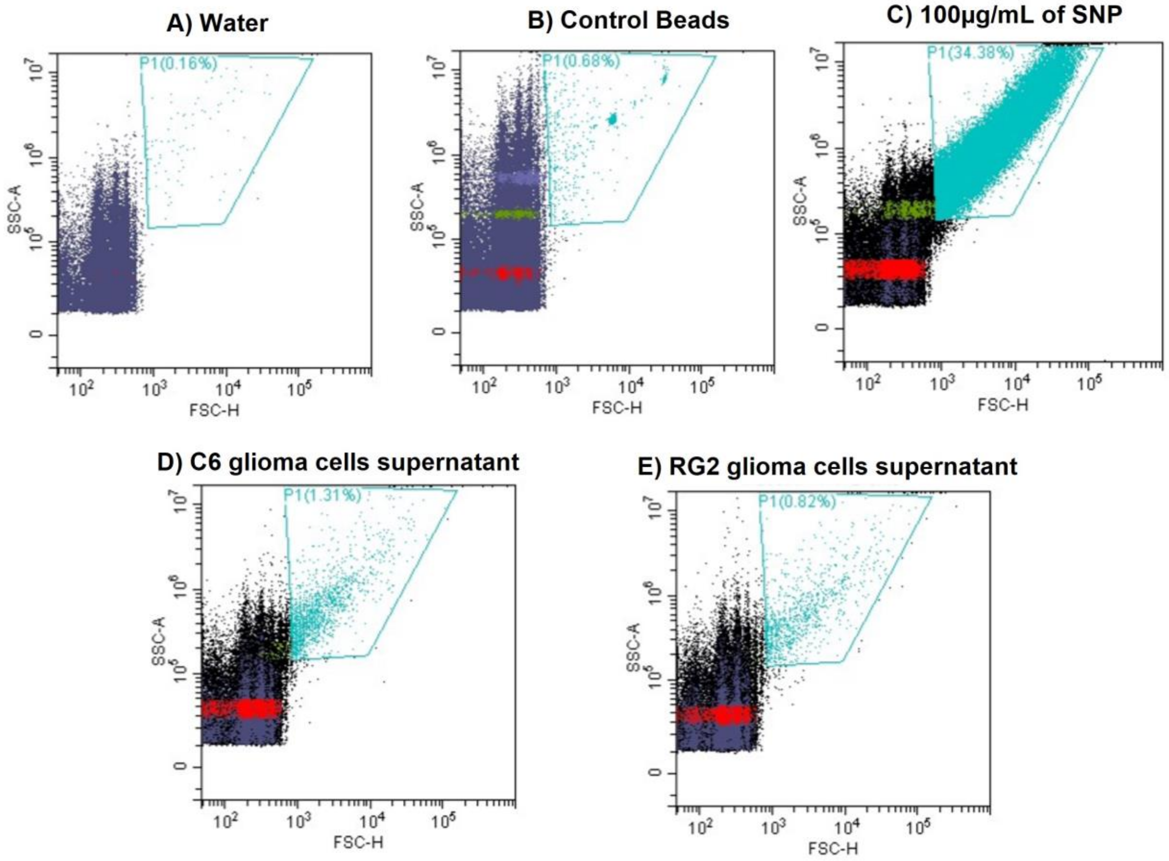

F)

Sample	Events	Percentage of SNPs in supernatant	NPs uptake	Uptake Percentage
Stock (100 µg/mL)	64465	100%		
C6 cells	18110±1882	28.0%	46355±1882	72%
RG2 cells	10660±2960	16.5%	53805±2960	83.5%

Figure S1. Cellular uptake of SNP by glioma cells. C6 and RG2 glioma cells were incubated for 24 h with 100 µg/mL SNP; next, cell supernatants were collected. The amount of NPS endocytosed by glioma cells was calculated by subtracting the amount of nanoparticles in the cell supernatants from the amount of nanoparticles in the stock of 100 µg/mL. Nanoparticles counting was performed using a CytoFlex SRT cell sorter and its violet laser (Beckman Coulter, USA). Figure shows representative dot plots of injectable water **(A)**, calibration control beads for the violet laser **(B)**, 100 µg/mL SNP stock **(C)**, supernatant of treated C6 cells **(D)** and supernatant of treated RG2 cells **(E).** Percentage of NPs uptake by C6 and RG2 cells. Results are presented as mean ± SD of one experiment by triplicate.
